# Investigation of Factors Influencing Formation of Nanoemulsion by Spontaneous Emulsification: Impact on Droplet Size, Polydispersity Index, and Stability

**DOI:** 10.3390/bioengineering9080384

**Published:** 2022-08-12

**Authors:** Mohammed S. Algahtani, Mohammad Zaki Ahmad, Javed Ahmad

**Affiliations:** Department of Pharmaceutics, College of Pharmacy, Najran University, Najran 11001, Saudi Arabia

**Keywords:** nanoemulsion, spontaneous-emulsification, vortexing time, droplet size and size distribution, stability, drug delivery

## Abstract

Interest in nanoemulsion technology has increased steadily in recent years for its widespread applications in the delivery of pharmaceuticals, nutraceuticals, and cosmeceuticals. Rational selection of the composition and the preparation method is crucial for developing a stable nanoemulsion system with desired physicochemical characteristics. In the present study, we investigate the influence of intricate factors including composition and preparation conditions that affect characteristic parameters and the stability of the nanoemulsion formation prepared by the spontaneous emulsification method. Octanoic acid, capryol 90, and ethyl oleate were selected to represent oil phases of different carbon–chain lengths. We explored the impact of the addition mode of the oil–S_mix_ phase and aqueous phase, vortexing time, K_m_ (surfactant/cosurfactant) ratio, and the replacement of water by buffers of different pH as an aqueous system. The phase behavior study showed that the S_mix_ phase had a significant impact on the nanoemulsifying ability of the nanoemulsions composed of oil phases of varying carbon-chain lengths. The mode of mixing of the oil–S_mix_ phase to the aqueous phase markedly influenced the mean droplet size and size distribution of the nanoemulsions composed of oil phases as capryol 90. Vortexing time also impacted the mean droplet size and the stability of the generated nanoemulsion system depending on the varying carbon-chain length of the oil phase. The replacement of the water phase by aqueous buffers of pH 1.2, 5.5, 6.8, and 7.4 has altered the mean droplet size and size distribution of the nanoemulsion system. Further, the K_m_ ratio also had a significant influence on the formation of the nanoemulsion system. The findings of this investigation are useful in understanding how the formulation composition and process parameters of the spontaneous emulsification technique are responsible for affecting the physicochemical characteristics and stability of the nanoemulsion system composed of oil of varying carbon-chain (C_8_-C_18_) length.

## 1. Introduction

Nanoemulsion (NE) has successfully carved out a niche amongst the plethora of other lipid-based nanoformulations (such as liposomes, solid lipid nanoparticles, and nanostructured lipid carriers) and polymeric nanoparticle formulations. This success is attributable to its small droplet size (10–200 nm), kinetic stability, optical clarity, and substantial ability to improve the dissolution and bioavailability of encapsulated active ingredients [1,2]. NEs are being explored for the delivery of important therapeutics [3,4], nutraceuticals [5], and bioactive compounds [6,7] through different routes of administration for various indications [8]. The preparation of the NE is mainly divided into two major methods: high-energy and low-energy emulsification [9,10]. The high-energy method utilizes devices such as high-pressure homogenizers and high-speed homogenizers to induce disruptive forces to the emulsion system. The more energy applied, the smaller droplets are obtained, allowing for the production of NE with higher oil/surfactant ratios. However, the need for mechanical devices and the high energy consumption to produce the nano-scaled droplets make it a costly and resource-consuming process. On the other hand, low-energy methods are simpler, more energy-efficient, and scalable where they utilize the internal chemical energy of the system components. These methods are classified based on whether the phase inversion of the curvature surfactant is produced or not during the emulsification process. Spontaneous emulsification, phase inversion temperature, and phase inversion composition are examples of such classifications [11].

In spontaneous emulsification, the organic phase that is constituted by oil, surfactant, and co-surfactant is simply added to the aqueous phase with mild agitation. The surfactant present in the organic phase has a high affinity for the continuous phase. Therefore, upon mixing of two phases (organic or dispersed phase and aqueous or continuous phase), turbulence is produced and surfactant diffuses swiftly towards the aqueous phase and forms covering/film around the dispersed oil droplet by lowering the interfacial tension resulting in the spontaneous formation of NE system. Co-surfactant further aids in causing the turbulence, lowering the interfacial tension between the two immiscible phases and easing the formation of dispersion by lodging in the unoccupied places around dispersed oil droplets that are left unguarded by surfactant molecules [12,13]. The screening of surfactants for the spontaneous emulsification is based on the hydrophilic–lipophilic balance (HLB) which is the strength and the size of the hydrophilic and lipophilic moieties of the surfactant molecule [14,15].

There have been studies that have hinted at the effect of the composition of oil and aqueous phase, temperature, pH, and stirring speed; however, still there is a substantial limitation to the access of the literature that establishes the detailed impact of parameters such as buffers (as a replacement to aqueous phase), Km, vortexing time, and mode of mixing of oil and aqueous phase on the droplet size of nanoemulsion prepared via spontaneous emulsification. A glimpse of the effect of the selected parameters was provided in different research studies but there has not been any study conducted so far that solely establishes their role on the characteristics of nanoemulsion. Therefore, this is the first study that provides the explicit investigation of the effect of essential parameters such as buffers (as a replacement to the aqueous phase), Km, vortexing time, and mode of mixing of oil and aqueous phase on the droplet size of nanoemulsion prepared via spontaneous emulsification. Importantly, critically and rationally optimized placebo formulation systems via investigating the effect of these selected parameters have the potential to contribute significantly to hastening the nanoemulsion-based product development for various applications (pharmaceutical, nutraceutical, and cosmeceutical) in future research.

Preceding research studies have demonstrated that the formation and properties of the NE prepared by spontaneous emulsification are dependent on the formulation and process variables. Saberi and associates have fabricated vitamin E-loaded NEs via the spontaneous emulsification method and their study outcome highlighted that several factors strongly impacted the formation and the properties of vitamin E-loaded NEs including the oil composition (vitamin E to medium-chain triglycerides ratio), surfactant concentration, aqueous and oil–S_mix_ phase mixing temperature and the stirring speed [16]. In another study by Komaiko and associates, surfactant to oil ratio, surfactant type, initial surfactant location, and the oil type were the governing factors that dictated the droplet size of NE prepared by the spontaneous emulsification method [17].

**Figure 1:**
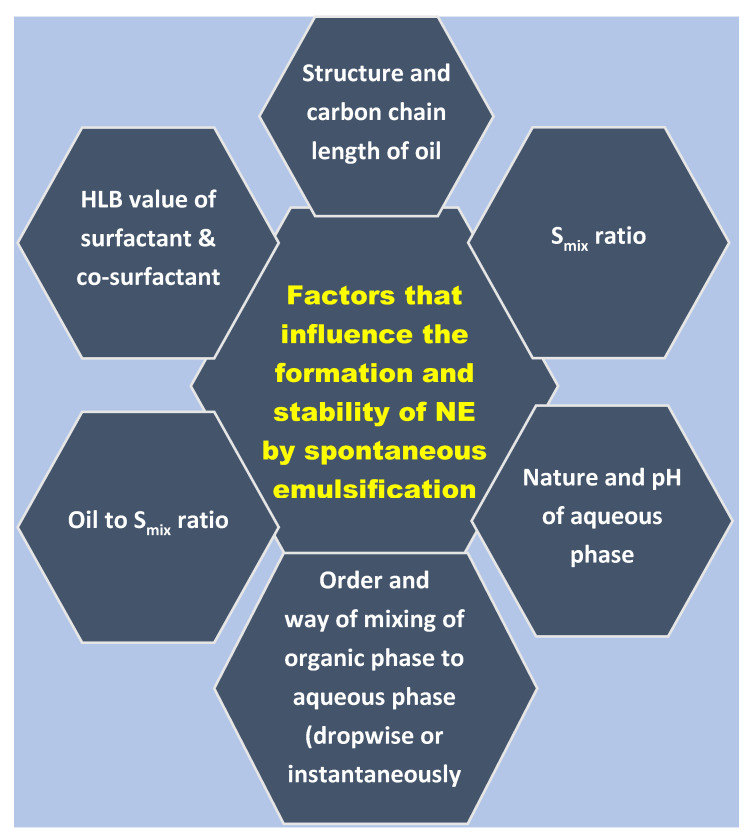
Schematic that shows the possible factors that affect the formation and the stability of NE via spontaneous emulsification.

Therefore, though this procedure of spontaneous emulsification appears simple, several crucial factors govern the characteristic properties and the stability of NEs formed by this low-energy approach. These may include the type and the structure of the oil phase, the emulsification ability of the surfactant and co-surfactant for different oil phases, the surfactant–cosurfactant mass ratio (K_m_ ratio), the mode of mixing of oil–Smix phase to the aqueous phase, vortexing time, and pH of an aqueous phase in replacement of water as an aqueous buffer. The possible factors that affect the formation and the stability of NE via spontaneous emulsification are illustrated in Figure 1. This work aims to study in-depth the impact of these factors on the formation and stability of NE prepared by the spontaneous emulsification process. 

## 2. Materials and Methods

### 2.1. Materials and Preparation of Different Buffers

Ethyl oleate, octanoic acid, Solutol HS15, and Cremophore EL were procured from Sigma-Aldrich, (St. Louis, MO, USA). Tween 80 and Tween 20 were obtained from Merck, Schuchardh, Hokenbrunn, Germany. Transcutol HP and Capryol 90 were procured from Gattefosse (Saint Priest, France). All chemicals and reagents used in the study were of pharmaceutical grade.

#### 2.1.1. Preparation of Hydrochloric Acid Buffer pH 1.2

For preparing an acidic buffer of pH 1.2, 50.0 mL of 0.2 M potassium chloride was placed in a 200 mL volumetric flask and 85 mL of 0.2 M hydrochloric acid was added, and then the volume was made up by adding water. 

#### 2.1.2. Preparation of Phosphate Buffer pH 5.5

Accurately weighed 13.61 g potassium dihydrogen phosphate was dissolved in distilled water and the volume was made up to 1000 mL (sol. A). Accurately weighed 35.81 g of disodium hydrogen phosphate was dissolved in water and the volume was made up to 1000 mL (sol. B). Then, 96.4ml of sol. A was mixed with 3.6 mL of sol. B.

#### 2.1.3. Preparation of Phosphate Buffer pH 6.8

To prepare phosphate buffer pH 6.8, 50 mL of 0.2 M potassium dihydrogen orthophosphate was placed in a 200 mL volumetric flask, 22.4 mL of 0.2 M sodium hydroxide was added, and then distilled water was added and the volume was made up. The pH was checked using a calibrated pH meter.

#### 2.1.4. Preparation of Phosphate Buffer pH 7.4

To prepare phosphate buffer pH 7.4, 50 mL of 0.2 M potassium dihydrogen orthophosphate was placed in a 200 mL volumetric flask, 39.1 mL of 0.2 M sodium hydroxide was added, and then distilled water was added and the volume was made up. The pH was checked using a calibrated pH meter.

### 2.2. Screening of Components

Screening of components, including oil phase, surfactant, and co-surfactant, is a crucial part of the preparation of NE. The oil phases were selected based on their varying chain length of carbon (C_8_-C_18_) atoms to study how this carbon chain length will impact the mean droplet size, polydispersity index (PDI), and stability of the oil-in-water (o/w) NE. The surfactant and co-surfactant were selected based on their emulsification abilities as elucidated in detail in the proceeding sections. 

#### 2.2.1. Selection of Oil

Oils of different carbon chain lengths (C_8_-C_18_) were utilized in the preparation of NE through the spontaneous emulsification method [16,18]. Octanoic acid (OA), capryol 90 (C90), and ethyl oleate (EO) were selected as model oil phases in this study. A description of the oil phase used in the current investigation is provided in the Supplementary Materials. 

#### 2.2.2. Selection of Surfactant

The emulsification ability of surfactants by the spontaneous emulsification method for the different oil phases was investigated [19]. Tween 20, Tween 80, Solutol HS15, and Cremophore EL were screened as surfactant phase. Surfactants were selected based on their HLB value, which should ideally be near 15. 

#### 2.2.3. Selection of Co-Surfactant

With the addition of a co-surfactant, the bending stress of the interface is reduced and the interfacial film is sufficiently flexible to adjust as per different curvatures required to form NE over a broad spectrum of composition [15,20]. Based on the wide reports in the literature and previous research experience, Transcutol HP (diethylene glycol monoethyl ether) was considered as a model co-surfactant. 

### 2.3. Emulsifying Efficiency of S_mix_ for OA, C90, and EO 

To evaluate the emulsifying efficiency of the S_mix_ phase, the S_mix_ was prepared by mixing 1 mL of 10% (*v*/*v*) surfactant and co-surfactant in a ratio of 1:1 and placed in 5 mL glass vials [21]. The 5 µL of the chosen oils (OA, C90, and EO) were added repeatedly with a gentle agitation into the aqueous solution of the S_mix_ phase until the sample mixtures turned turbid. The transparent samples were allowed to equilibrate for about 24 h followed by a visual examination for optical clarity [22,23]. 

#### Determination of Percentage Transmittance (%T)

After the transparent samples were allowed to equilibrate, they were tested for %T using a UV spectrophotometer (Shimadzu, Kioto, Japan) at λmax 638.2 nm to assess the optical clarity and emulsifying ability [15]. The measurements were done in triplicate. 

### 2.4. Phase Behavior Study

The surfactant with the maximum emulsifying ability for oils was selected for phase behavior studies for a specific combination of the S_mix_ phase. The ratio of the surfactant and co-surfactant mixture (S_mix_) was 1:1 based on pre-experimental trials. The phase diagrams of the pseudo-ternary system (oil phase, S_mix_ phase, and aqueous phase) were constructed using the aqueous titration (spontaneous emulsification) method [15,20]. An adequate quantity of S_mix_ was dissolved in different oil phases in glass vials at room temperature. Each oil–S_mix_ mixture was titrated drop-wise continuously with double distilled water using a micropipette by vortex mixing until it turned turbid. The changes in the clarity of the preparation during the titration were diligently observed. The percentage composition of the component in each pseudoternary system was determined and the observed results were plotted on triangular coordinates to construct the phase diagrams. 

#### 2.4.1. Determination of Area of Nanoemulsification Region

The calibration plot method was used to determine the region of the nanoemulsification in the developed phase diagram. The calibration plot was constructed between the weight (mg) of graph paper versus its area (cm^2^). The nanoemulsification region was cut and sketched on graph paper used in the construction of the calibration plot. The area sketched on graph paper was cut and weighed and the corresponding area was determined from the regressed equation of the calibration plot [15].

### 2.5. Preparation and Optimization of the NE Formulation by Spontaneous Emulsification Method

The NEs were prepared in different compositions from the oil phase as OA, C90, and EO at a S_mix_ mass ratio (K_m_) of 1:1 of different concentrations obtained from nanoemulsification regions found through phase behavior study [16,18].

The mixing oil–S_mix_ phase to the aqueous phase and vice-versa was performed in four different modes: (i) Instantaneous mixing of aqueous phase to oil–S_mix_ phase; (ii) Drop-by-drop mixing of aqueous phase to oil–S_mix_ phase; (iii) Instantaneous mixing of oil–S_mix_ phase to the aqueous phase, and (iv) Drop-by-drop mixing of oil–S_mix_ phase to the aqueous phase. 

All modes of mixing were subjected to vortexing for 0, 1, 3, and 5 min to investigate the impact of vortexing time on the mean droplet size, PDI, and the stability of optimized NE (schematic illustration in Supplementary Figure S1).

The impact of the addition of an aqueous buffer system with oil–S_mix_ as a replacement for the water on the mean droplet size and PDI was also evaluated. The oil–S_mix_ was mixed with aqueous buffers of different pHs 1.2, 5.5, 6.8, and 7.4. 

Furthermore, the impact of the S_mix_ mass ratio (K_m_) on the characteristic features of NE was also investigated by preparing the S_mix_ phase of NE in different mass ratios (such as 1:1.5, 1:2, 1:3, 1:4, 1.5:1, 2:1, 3:1, and 4:1).

#### Mean Droplet Size and PDI Determination

The mean droplet size and size distribution in form of PDI of the diluted (1:100) NE were determined by the dynamic light scattering technique using Zetasizer (Malvern Instruments, 1000 HS, Malvern, UK) [24] to observe the influence of the mode of preparation method and different process parameters.

### 2.6. Stability Testing of NE System 

The storage stability of optimized NE systems composed of OA, C90, and EO as oil phase were evaluated. Samples of different oil–S_mix_ ratios (1:1.5, 1:1.75, 1:2, 1:2.5, 1:3, 1:3.5, and 1:4) were prepared in Eppendorf tubes and stored for 1 month at 25 °C. The effects of storage conditions on the physical appearance, mean droplet size, and PDI of samples were evaluated at time intervals of 0, 15, and 30 days [25,26].

### 2.7. Statistical Analysis

All of the presented results are expressed as the mean ± standard deviation (SD). Statistical comparison of results between different groups was performed using the unpaired *t*-test and one way ANOVA (GraphPad Prism version 9.3.1.471 for window, GraphPad Software, San Diego, CA, USA), where *p* < 0.05 was considered a significant difference between the compared groups.

## 3. Results and Discussion

### 3.1. The Emulsifying Efficiency of the Smix Phase for Different Oil Phases

The emulsification ability of different S_mix_ mixtures (tween 20, tween 80, solutol HS15, cremophor EL individually mixed with transcutol HP in 1:1) was tested for oil phases of varying carbon chain length (C_8_-C_18_ such as OA, C90, and EO) and lipophilicity. The results are presented in Table 1. As discussed above, the selection of transcutol HP was based on previous literature and promising experimental results [15,20,27,28]. The emulsification ability was assessed based on the volume of that particular oil emulsified [21] in the S_mix_ phase and the formation of clear dispersion without being very hazy in appearance. The clarity of formed dispersion was standardized by determining %T which should not be less than 80% [19]. 

In general, the S_mix_ phase was prepared to have more emulsification efficiency for OA than the oil phase, followed by the C90 and EO, respectively. For all three types of oil phases, the S_mix_ phase of cremophore EL showed the maximum emulsification efficiency followed by the S_mix_ phase of tween 80 and then tween 20, respectively, while the S_mix_ phase of solutol HS15 showed minimum emulsification efficiency (Table 1). Although the utilization of cremophore EL in the pharmaceutical formulation is common at specified concentration and its safety limit is already defined by the regulatory agency for human use in pharmaceutical or nutraceutical products. It was observed that samples of all these tested dispersion systems exhibited considerable % transmittance (>80%) as shown in Table 1. 

The reason for the higher solubilization efficiency of the S_mix_ phase for OA as an oil system is attributed to the fact that it has shorter carbon chain and lower lipophilicity (HLB value approaching 10) compared with C90 and EO, which has a longer carbon chain and higher lipophilicity (HLB value approaching towards 1) [14]. Therefore, the length of the carbon chain needs to be taken into consideration while designing a NE system, as the increase in the carbon chain length of the oil phase reduces the emulsification ability of the surfactant/S_mix_ phase. 

### 3.2. Pseudoternary Phase Diagram Study

**Figure 2:**
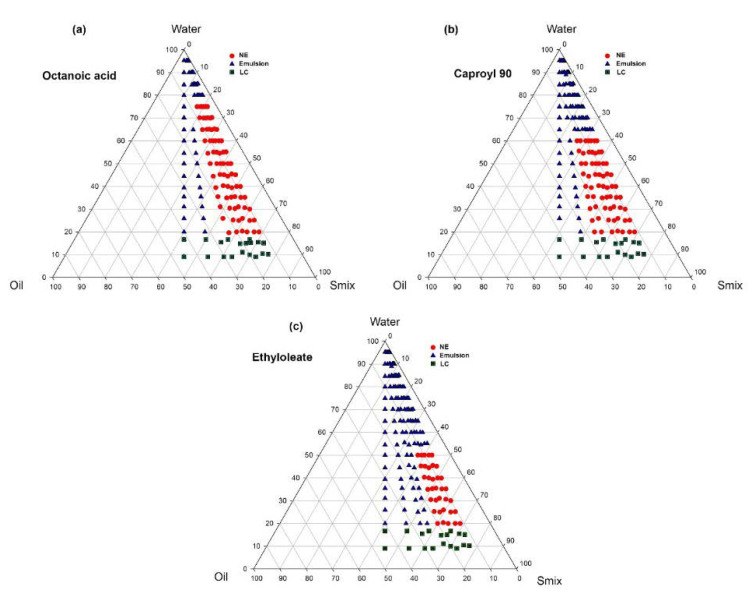
Phase behavior study by constructing pseudoternary phase diagrams for oil phase consisting of (**a**) octanoic acid as oil phase at Smix ratio 1:1, (**b**) capryol 90 as oil phase at Smix ratio 1:1, and (**c**) ethyl oleate as oil phase at Smix ratio 1:1.

The phase behavior study was performed according to the procedure described in Section 2.4 and the NE region was calculated as per the procedure elaborated in Section 2.4.1. The NE region obtained in Figure 2 depicts that the maximum NE region obtained in the case of colloidal dispersion consists of OA as the oil phase. The size of the NE region obtained in the phase behavior study follows the order of OA > C90 > EO. 

The HLB value of the oil phase consisting of OA is greater than the HLB value of the oil phase consisting of EO, signifying that the low lipophilicity of the oil phase consisting of OA [15] has more affinity for the aqueous system as compared with C90 and EO. It is ultimately responsible for the greater NE region obtained in the phase behavior study in the case of OA compared to EO. The phase diagrams (Figure 2) depict that the chosen S_mix_ concentration was able to considerably increase the dispersion entropy, reduce the interfacial tension, increase the interfacial area and lower the free energy of the system to a minimum possible value, resulting in the formation of substantial NE regions for the oil phase consist of OA, C90, and EO [14,29]. 

The pseudoternary diagrams depict the influence of each component (oil, S_mix,_ and water) in the formation and stabilization of the formulated NE prepared through the spontaneous emulsification method [22,23]. It has been observed that the greater the HLB value of the S_mix_ system, the greater its emulsification efficiency; the HLB value of Cremophore EL is approximately 13.5 and that of Tanscutol HP is 4.2. The role of Transcutol HP as a co-emulsifier along with surfactant having HLB between 12 to 15 in the preparation and stability of NE is well established. The presence of Transcutol HP also contributes significantly to the formation of a stable colloidal dispersion of NE by imparting flexibility to the surfactant film and overcoming the repulsive forces and fluidity of the respective aqueous and oil phases [15,20,27,28]. 

The HLB value is the dictating factor in governing the amalgamation of the aqueous and oil phases in the course of the transformation into the NE system. Therefore, a greater HLB value in the surfactant system would have imparted more hydrophilicity and affinity towards the aqueous phase, resulting in optimal NE regions for colloidal dispersion system consisting of OA, C90, and EO as oil phase. [17,29].

### 3.3. Preparation and Optimization of NE Formulation by Spontaneous Emulsification Method

Series of NEs were prepared by the spontaneous emulsification method as described in Section 2.5. The droplet size, PDI, and %T were determined for different formulations compositions of %oil, %S_mix,_ and %water as shown in Table 2. These key parameters of NE are going to affect the bulk properties, product performance, appearance, and stability [30]. 

Out of the six formulations formulated for each oil type (Table 2), five formulations from OA as oil phase, three formulations from C90 as oil phase, and two formulations from EO as oil phase had a droplet size below 100 nm (Table 2). The smallest droplet size (20.45 nm) was obtained from the formulation consisting of 5.5% OA, 45.33% S_mix,_ and 49.11% water. Despite the use of the same oil% (5.5) of OA, the droplet size nearly doubled (38.78 nm) when the S_mix_ decreased to 9.45%. The same was noticed for NE formulations consisting of 26.67% oil of OA, where the droplet size significantly (*p* < 0.05) decreased from 177.7 nm to 47.65 nm when the S_mix_% increased from 9.45% to 45.33%.

In the case of C90 as the oil phase, the impact of S_mix_% on the droplet size is significant. For example, in NE formulations that consist of 16.67 % of oil, the increase in the S_mix_ % from 35.33% to 46.51% has resulted in an increase in droplet size from 34.47 nm to 58.98 nm and get unstable after 24h. Similarly for NE formulations having 23.26% of the oil phase of C90, the increase in the S_mix_% from 35.33% to 46.51% has resulted in a significant (*p* < 0.05) decrease in droplet size from 136.9 nm to 48.37 nm and remained stable even after 24 h (Table 2). 

In the case of EO as oil phase, the increase of S_mix_% resulted in a decrease in the droplet size. For example, in NE formulations that consist of 13.7% of oil, the increase in the S_mix_% from 41.10 to 56.07% resulted in a significant (*p* < 0.05) decrease in droplet size from 919.9 nm to 29.69 nm and remained stable after 24h (Table 2). Similarly, for NE formulations that have 16.20% of oil, the increase in the S_mix_% from 48.58% to 56.07% resulted in a significant (*p* < 0.05) decrease in droplet size from 127.5 nm to 28.04 nm and remained stable after 24 h (Table 2). 

For the impact of S_mix%_ on the PDI, results showed that an increase in the S_mix_% resulted in an increase in PDI in the case of NE consisting of OA and EO as oil phase. For example, in NE formulations that consist of 5.56% of the oil phase of OA, the increase in the S_mix_% from 9.45% to 45.33% resulted in a significant (*p* < 0.05) increase in PDI from 0.210 to 0.321. Similarly, for NE formulations that have 26.67% of oil of OA, the increase in the S_mix_% from 9.45% to 45.33% has resulted in a significant (*p* < 0.05) increase in PDI from 0.125 to 0.497 (Table 2). In addition, in NE formulations that consist of 13.7% of the oil phase of EO, the increase in the S_mix_% from 41.10% to 56.07% resulted in a significant (*p* < 0.05) increase in PDI from 0.135 to 0.336, while in NE formulations that have 18.69% of the oil phase of EO, the increase in the S_mix_% from 41.10% to 56.07% resulted in a significant (*p* < 0.05) increase in PDI from 0.383 to 0.589 (Table 2). 

In the case of C90 as the oil phase, the increase of S_mix_% resulted in a decrease in the PDI. For example, in NE formulations that consist of 16.67% of the oil phase of C90, the increase in the S_mix_% from 35.33% to 46.51% resulted in a decrease in PDI from 0.093 to 0.072 while in NE formulations that have 23.26% of the oil phase of C90, the increase in the S_mix_% from 35.33% to 46.52% has resulted in significant (*p* < 0.05) decrease in PDI from 0.527 to 0.056 (Table 2). 

Out of the six NE formulations prepared for each oil type (Table 2), five formulations from OA as oil phase, three formulations from C90 as oil phase, and only two formulations from EO as oil phase demonstrated stability after 24 h. In NE formulations consisting of OA as oil phase, stability was affected when the S_mix_% increased from 27.39% to 45.33% in only one case of NE consisting of 16.11% oil (Table 2). In NE formulations consisting of C90 as the oil phase, the impact of S_mix_% on the two NE formulations having the same oil% was quite different. For example, in the case of an NE formulation that has 16.67% of oil, the increase in the Smix% from 35.33% to 46.51% resulted in an unstable NE formulation while in NE formulations that have 23.26% of oil, the increase in the S_mix_% from 35.33% to 46.51% has not affected the formulation stability (Table 2). In NE formulations consisting of EO as oil phase, an increase of S_mix_% from 41.10% to 56.07% was needed to obtain a stable NE consisting of 13.7% of oil. Further, in the formulation consisting of 16.20% of oil, an increase of S_mix_% from 48.58% to 56.07% was needed to obtain a stable NE system.

S_mix_% has shown a remarkable impact on the droplet size, PDI, and stability of NE. The increase in the S_mix_% is expected to decrease the droplet size in the case of OA, C90, and EO as the oil phase. Further, the increase in the S_mix_% is expected to increase the PDI in NE consisting of OA and EO as oil phase, while expected to decrease the PDI in NE consisting of C90 as oil phase. The stability of NE formulation might be affected when the S_mix_% increased in the same amount of oil%. Our results clearly show that the stability behavior of NE formulation consisting of oil phase as OA, C90, and EO is quite different. The reason behind such an observation could be the increased lipophilicity with the increase in the length of the carbon chain which would require greater energy or S_mix_ concentration to lower the interfacial tension and generate a stable NE system [31,32]. However, these observations also suggest that the increase in S_mix_ concentration beyond a critical point at which the preparations are highly stabilized would lead to disruption of the stability and therefore, excess S_mix_ concentration should be avoided [33,34]. 

There is a negative correlation between %T and the mean droplet size of OA-based, C90-based, and EO-based NE systems with R^2^ > 0.99 as shown in Supplementary Figure S2. The correlation shows that the decrease in the %T is associated with the increase in the droplet size, which corroborates results from previous literature [15,29]. 

### 3.4. The Impact of Different Variables on the Mean Droplet Size, PDI, and the Stability of NE

For investigation of the impact of different formulation and process factors on the mean droplet size, PDI and stability of NE system composed of OA, C90, and EO as oil phases were freshly prepared and investigated. 

#### 3.4.1. Mode of Mixing of Aqueous Phase to Oil–S_mix_ Phase and Vice-Versa

Four different modes of mixing were used to prepare NE systems containing OA, C90, and EO as oil phases, including: (i) Instantaneous mixing of aqueous phase to oil–S_mix_ phase; (ii) Drop-by-drop mixing of aqueous phase to oil–S_mix_ phase; (iii) Instantaneous mixing of oil–S_mix_ phase to the aqueous phase, and (iv) Drop-by-drop mixing of oil–S_mix_ phase to the aqueous phase. The effect of mode of mixing of aqueous to oil–Smix and vice versa was investigated for (1) NE system composed of OA as oil to Smix ratio 1:3 and optimized composition as 300 µL oil phase, 900 µL Smix phase, and 800 µL water; (2) NE system composed of C90 as oil to Smix ratio 1:3 and optimized composition as 300 µL oil phase, 900 µL Smix phase, and 800 µL water; and (3) NE system composed of EO as oil to Smix ratio 1:4 and optimized composition as 300 µL oil phase, 1200 µL Smix phase, and 500 µL water. From the results presented in Table 3, it can be seen that mode of mixing of NE components had no significant (*p* > 0.05) influence on mean droplet size, PDI, and %T in the case of OA-based NE and EO-based NE. However, in the case of C90, the NEs prepared by drop-wise mixing mode exhibited significantly (*p* < 0.05) smaller droplet size as compared to those NEs prepared by instantaneous mixing of aqueous phase to oil–S_mix_ phase (nanoemulsion formulations prepared through drop-wise mixing mode are shown in Supplementary Figure S2). Therefore, drop-by-drop mixing is preferable to get a smaller droplet size in the case of NE consisting of a C90-based NE system.

#### 3.4.2. Impact of Vortexing Time on the Droplet Size, PDI, and the Stability of the NE

**Figure 3:**
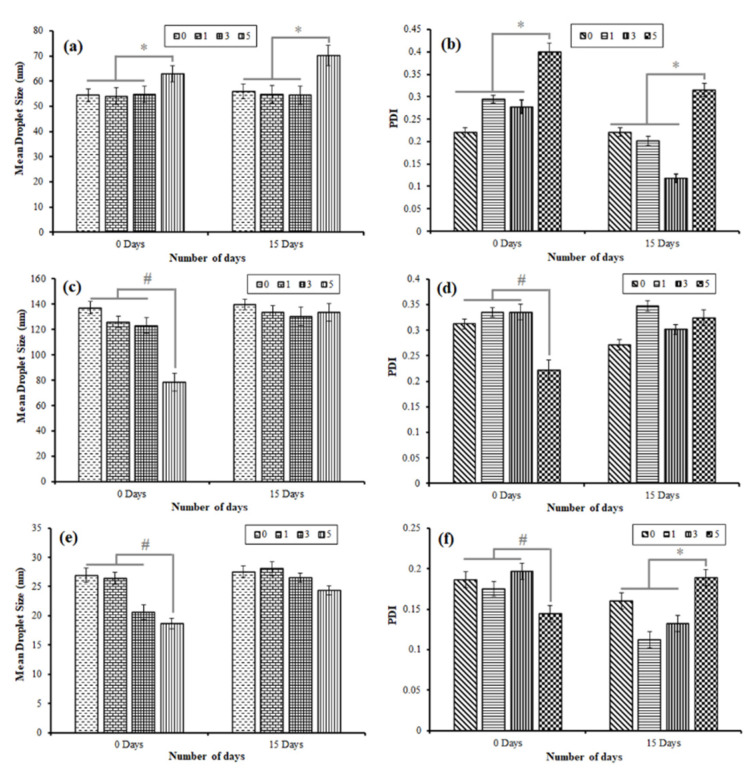
Impact of vortexing duration (0, 1, 3, and 5 min) (**a**) on droplet size for OA-based NE; (**b**) on PDI for OA-based NE; (**c**) on droplet size for C90-based NE; (**d**) on PDI for C90-based NE; (**e**) on droplet size for EO-based NE; (**f**) on PDI for EO-based NE. * Increase is statistically significant (*p* < 0.05) with the increase of the vortexing duration; **#** Decrease is statistically significant (*p* < 0.05) with the increase of the vortexing duration.

To investigate the impact of vortexing duration, NEs of oil phases OA and C90 were prepared with 300 µL oil phase, 900 µL S_mix_ phase, and 800 µL water. NE of oil phase as EO was prepared with 300 µL oil phase, 1200 µL S_mix_ phase, and 500 µL water. A higher concentration of the S_mix_ was needed to prepare the NE containing EO, as the oil phase because of the high lipophilicity of EO (low HLB value) compared to the OA and C90. The freshly prepared NE systems were vortexed for 0 (no vortexing), 1, 3, or 5 min. The impact of the vortexing time on the droplet size, and PDI (storage duration 0, and 15 days) of the OA-based, C90-based, and EO-based NE systems is presented in Figure 3a–f. 

The influence of vortexing duration was found to be directly related to the mean droplet size and PDI in the case of the OA-based NE system, where the increase in mean droplet size and PDI were statistically significant (*p* < 0.05) with the increase of the vortexing duration (Figure 3a,b). On another side, vortex’s duration was found to be inversely related to mean droplet size and PDI in the case of C90-based, and EO-based NE systems, where the mean droplet size and PDI were significantly (*p* < 0.05) decreased with the increase of the vortexing duration (Figure 3c–f). 

Interestingly, the stability profile (any changes observed in mean droplet size and PDI of the NE system upon storage for 15 days) of all three types (OA-based, C90-based, and EO-based) of the NE system are affected almost to an equal extent by increasing the duration of vortexing. The stability of the prepared NE system was consistent under no vortexing conditions. However, following 15 days of storage, the values of mean droplet size and PDI remained relatively constant for OA-based NE for vortex times of 1 and 3 min, whereas these values increased slightly for C90-based and EO-based NE systems for vortex durations of 1 and 3 min. The increase in the duration of vortexing up to 5 min resulted in a significant (*p* < 0.05) increase in mean droplet size and PDI of OA-based, C90-based, and EO-based NEs after 15 days of storage. These results indicate that the stability of all the freshly prepared NE systems by spontaneous emulsification is greatly influenced by the increase in vortex duration upon storage for 15 days. The mean droplet size and PDI of the freshly prepared NE system no longer remained constant upon storage. The study results suggest that preparation of the NE system by spontaneous emulsification without vortexing is a preferable condition for maintaining longer stability of the NE system consisting of OA, C90, and EO as oil phase (Figure 3a–f).

#### 3.4.3. Effect of the Buffer as Aqueous Phase (pH 1.2, 5.5, 6.8, 7.4)

The use of buffer as the aqueous phase in NE holds significant importance, particularly in the case of sensitive drug candidates, such as ramipril, which is very sensitive to the pH of the aqueous phase and is prone to degradation in the presence of moisture and alkaline pH [35,36]. In a research study conducted by Shafiq et al., the aqueous phase of NE was replaced by a standard buffer of pH 5, which significantly affected the stability of ramipril loaded in the NE system [24]. Therefore, in the present investigation, the impact of buffers of different pHs on the mean droplet size and PDI of the NE system was explored to decipher how different pH values (1.2, 5.5, 6.8, and 7.4) affect the characteristic response parameter of the NE system composed of oil phases of different carbon chain lengths, such as OA, C90, and EO. The NEs of oil phases such as OA and C90 were prepared with 300 µL oil phase, 900 µL S_mix_ phase, and 800 µL water. The NE of oil phase as EO was prepared with 300 µL oil phase, 1200 µL S_mix_ phase, and 500 µL water.

**Figure 4:**
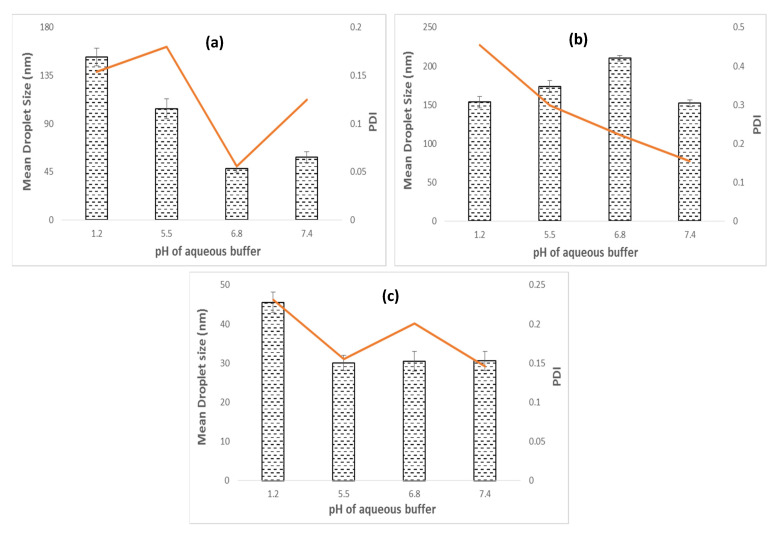
Impact of buffers of different pH on mean droplet size and PDI of NE system composed of (**a**) OA as oil phase; (**b**) C90 as oil phase; (**c**) EO as oil phase.

It was observed that the impact of buffers of different pH as an aqueous phase influences the mean droplet size and PDI of the NE system consisting of OA, C90, and EO as oil phases (Figure 4). The effect of the buffer as an aqueous phase was more pronounced, particularly in the case of OA-based NE. The smallest mean droplet size (48.07 nm) and low PDI (0.056) were observed at pH 6.8 in the NE system consisting of OA as the oil phase (Figure 4a). It was observed that the mean droplet size (152.1 nm) and PDI (0.154) of OA-based NE system was significantly increased in the buffer of acidic pH 1.2 compared to NE system at pH 6.8 (*p* < 0.05) (Figure 4a). OA-based NE formulation contains a carboxyl group as a pH-responsive group, which upon pH changes resulted in the protonation or deprotonation of the responsive group in the formulation system [37]. A pKa value of 4.89 of OA indicates that it will exist almost entirely in the anion form at pH values of 5–9 [38] and get protonated upon a change in pH to acidic pH 1.2. This switching of the responsive group between deprotonated to protonated state due to the change in pH of the aqueous phase is responsible for contributing to the pH-responsive nature of the NE [37] and results in an increase in mean droplet size of OA-based NE formulation at pH 1.2. On the contrary, it was observed that the mean droplet size (210.1 nm) of C90-based NE system was significantly larger in a buffer of pH 6.8 compared to the mean droplet size (153.5 nm) of C90-based NE system at pH 1.2 (*p* < 0.05) (Figure 4b). In addition, the impact of buffers of different pH (5.5, 6.8, and 7.4) on the change in mean droplet size of EO-based NE systems was not statistically significant (*p* > 0.05). Indeed, the largest mean droplet size (45.53 nm) and maximum PDI (0.231) were observed in the case of EO-based NE system consisting of a buffer of pH 1.2 as an aqueous phase (Figure 4c).

The result suggested that, in the NE system having OA, C90, and EO as the oil phase, the smallest droplet size and low PDI can be achieved by replacing water as the aqueous phase with the buffers of optimum pH as per the suitability of applications.

#### 3.4.4. Effect of K_m_ Ratio (Surfactant Co-Surfactant Mass Ratio)

**Figure 5:**
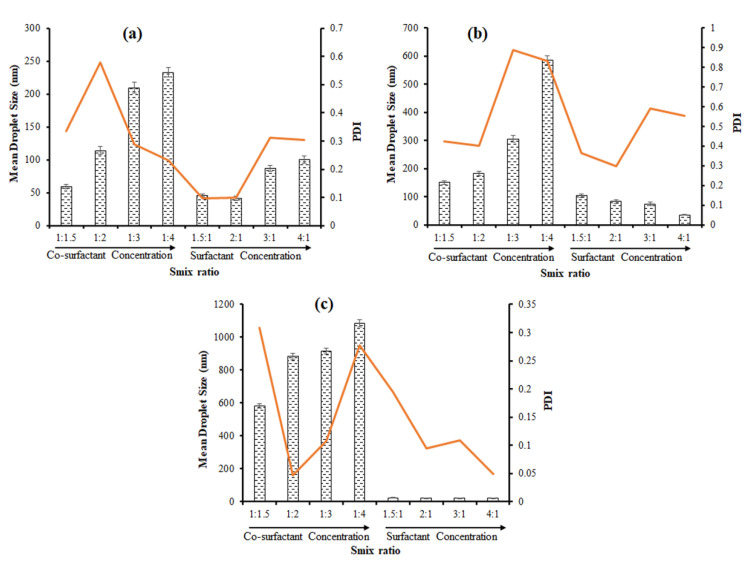
Impact of Km ratio on mean droplet size and PDI of NE system composed of (**a**) OA as oil phase; (**b**) C90 as oil phase; (**c**) EO as oil phase.

It is of note that the difference in K_m_ ratio significantly influences mean droplet size and PDI in the case of the NE system consisting of OA, C90, and EO as oil phases (Figure 5). The NEs of oil phases such as OA and C90 were prepared with 300 µL oil phase, 900 µL S_mix_ phase, and 800 µL water. The NE of oil phase as EO was prepared with 300 µL oil phase, 1200 µL S_mix_ phase, and 500 µL water. The K_m_ ratio effect is the most pronounced in the case of the NE system composed of EO as the oil phase.

In the case of the OA-based NE system, it was evident that the increase in the co-surfactant concentration in the K_m_ ratio gradually increases the mean droplet size (Figure 5a). The smallest mean droplet sizes were observed when the surfactant concentration was slightly higher than the co-surfactant concentration in the K_m_ ratios of 3:2 and 2:1 (Figure 5a). Interestingly, the mean droplet size increases with the increase of surfactant concentration to the co-surfactant concentration in the K_m_ ratios 3:1 and 4:1 (Figure 5a). The smallest droplet size (41.02 nm) and low PDI (0.096) were achieved at an optimal K_m_ ratio of 2:1 in the case of the OA-based NE system (Figure 5a).

The impact of the K_m_ ratio on mean droplet size and PDI was significant in the case of the C90-based NE system. The mean droplet size and PDI were significantly (*p* < 0.05) increased from (150.3 to 585.0 nm) when the co-surfactant concentration increased gradually to the surfactant concentration in K_m_ ratios 1:1.5 to 1:4 (Figure 5b). Conversely, the mean droplet size and PDI decreased significantly (*p* < 0.05) when the surfactant concentration gradually increased to the co-surfactant concentration in K_m_ ratios (1.5:1 to 4:1) (Figure 5b). The smallest mean droplet size (35.7 nm) was observed with the K_m_ ratio of 4:1 and the lowest PDI (0.298) was observed with the K_m_ ratio of 2:1 in the case of the C90-based NE system (Figure 5b).

Further, the impact of the K_m_ ratio was significant regarding the mean droplet size and PDI in the case of the EO-based NE system. The mean droplet size and PDI were significantly (*p* < 0.05) decreased to reach 20.71 nm and 0.046, respectively, when the surfactant concentration increased to the co-surfactant concentration in the K_m_ ratios 3:2 to 4:1 (Figure 5c). Conversely, there was a gradual increase in the mean droplet size and PDI when the co-surfactant concentration increased to the surfactant concentration in the K_m_ ratios 1:1.5 to 1:4 (Figure 5c). The smallest droplet size (20.71 nm) and the lowest PDI (0.046) were found at the K_m_ ratio of 4:1. In addition, the K_m_ ratio (1:2) minimizes the PDI (Figure 5c). 

The study findings indicate that the mean droplet size and PDI of OA-based, C90-based, and EO-based NE systems are greatly influenced by the K_m_ ratio and the concentration of the surfactant plays a major role in the reduction of the mean droplet size compared to the concentration of the co-surfactant. Su et al. has also reported the influence of the K_m_ ratio in reducing the mean droplet size of NE prepared through the phase-inversion composition (PIC) technique [39]. 

### 3.5. Stability of NE System Composed of OA, C90, and EO as Oil Phase 

The stability study of the NE system composed of OA, C90, and EO as oil phases was performed as per the procedure provided in Section 2.6. The NE formulations of all three categories of oil phases were prepared at a specific oil–S_mix_ ratio. The results of the stability study revealed that the NE system composed of OA as oil phase (with oil–S_mix_ ratio 1:3; 1:3.5, and 1:4) remained consistent (in respect of mean droplet size, PDI, and %T) throughout the 30 days storage duration (Table 4). 

The stability profile of the NE system composed of C90 and EO as oil phases was found to remain consistent during the 30 days storage duration at oil–S_mix_ ratios of 1:3 and 1:4, respectively (Table 4).

At the oil–S_mix_ ratio of 1:3, the NE system composed of OA and C90 showed consistent results in terms of mean droplet size, PDI, and %T, whereas a significant (*p* < 0.05) change in these physical attributes was observed in the case of the NE system composed of EO (Table 4) [40]. 

At the oil–S_mix_ ratio of 1:2 and 1:2.5, the NE system composed of all of the three oil phases (OA, C90, and EO) showed significant (*p* < 0.05) changes in the mean droplet size and PDI during the 30 days storage duration. The results of the stability study investigation support and align with the literature report that NE composed of OA-based, and C90-based NE systems were found to be more stable in comparison to EO-based NE systems [41,42,43,44].

## 4. Conclusions

The study demonstrates important observations regarding the formation and the characteristic features of NE systems composed of oil phases of varying carbon chain (C_8_-C_18_) lengths prepared by the spontaneous emulsification method. The vortexing duration, mode of addition of oil–S_mix_ to the aqueous phase, the replacement of water phase with aqueous buffers, and K_m_ value have substantially affected the characteristic features of the NE such as mean droplet size and size distribution. Furthermore, the stability profile of the NE system composed of OA and C90 had consistent results in terms of mean droplet size, PDI, and %T at a lower oil–S_mix_ ratio compared to the NE system composed of EO. This is due to the low lipophilicity (higher HLB value) of OA, and C90 as an oil phase compared to the EO as an oil. Taken together, these data have to be implemented to broaden the preformulation understanding on NE systems prepared through spontaneous emulsification technique and helpful to hasten the NE-based product development for different.

## Figures and Tables

**Table 1:** Volume of oil (OA, C90, and EO) emulsified and % transmittance (%T) of dispersion system consisting of oil emulsified in 10% aqueous Smix (1:1) system.

Oil Phase	Type of Smix	Vol. of Oil Emulsified (µL)	%T ± SD
Octanoic acid (OA)	Tween 20 and transcutol HP	30.0	88.84 ± 0.366
	Tween 80 and transcutol HP	40.0	83.83 ± 0.660
	Solutol HS15 and transcutol HP	28.0	81.89 ± 0.606
	Cremophore EL and transcutol HP	70.0	84.26 ± 0.113
Capryol 90 (C90)	Tween 20 and transcutol HP	14.0	88.74 ± 0.581
	Tween 80 and transcutol HP	15.0	87.95 ± 0.890
	Solutol HS15 and transcutol HP	9.0	87.75 ± 0.711
	Cremophore EL and transcutol HP	24.0	88.74 ± 0.911
Ethyl oleate (EO)	Tween 20 and transcutol HP	7.0	85.74 ± 1.101
	Tween 80 and transcutol HP	11.0	81.83 ± 1.807
	Solutol HS15 and transcutol HP	9.0	85.02 ± 1.378
	Cremophore EL and transcutol HP	21.0	87.70 ± 1.850

**Table 2:** Percentage composition of NEs prepared by spontaneous emulsification method and the impact on the formulation droplet size, PDI, and the stability (mean ± SD).

Oil Phase	%Oil	%Smix	%Water	%T	Size (nm)	PDI	Stability (24 h)
OA	5.56	9.45	84.99	91.25 ± 2.76	38.78 ± 0.15	0.210 ± 0.03	Stable
	26.67	45.33	28.0	98.33 ± 1.96	47.65 ± 0.43 ^a^	0.497 ± 0.02	Stable
	5.56	45.33	49.11	96.94 ± 0.68	20.45 ± 0.07	0.321 ± 0.05	Stable
	26.67	9.45	63.88	81.23 ± 1.24	177.7 ± 1.95 ^a^	0.125 ± 0.04	Stable
	16.11	27.39	56.5	98.01 ± 0.94	56.35 ± 0.39	0.172 ± 0.02	Stable
	16.11	45.33	38.56	98.23 ± 2.06	46.90 ± 0.21	0.111 ± 0.04	Unstable
C90	16.67	35.33	48.0	97.43 ± 1.73	34.47 ± 0.18	0.093 ± 0.001	Stable
	23.26	46.51	30.23	98.99 ± 0.88	48.37 ± 0.66 ^b^	0.056 ± 0.003	Stable
	16.67	46.51	36.82	93.61 ± 1.65	58.98 ± 0.72	0.072 ± 0.003	Unstable
	23.26	35.33	41.41	97.39 ± 1.79	136.9 ± 2.75 ^b^	0.527 ± 0.03	Stable
	19.96	45.92	34.12	83.01 ± 0.53	116.9 ± 1.05	0.550 ± 0.01	Unstable
	19.96	46.51	33.53	94.51 ± 1.01	142.7 ± 1.64	0.537 ± 0.04	Unstable
EO	13.70	41.10	45.2	15.29 ± 0.08	919.9 ± 23.93	0.135 ± 0.05	Unstable
	18.69	56.07	25.24	10.26 ± 0.03	194.17 ± 2.71	0.589 ± 0.04	Unstable
	13.70	56.07	30.23	97.28 ± 1.26	29.64 ± 0.04	0.336 ± 0.05	Stable
	18.69	41.10	40.21	10.35 ± 0.18	104.4 ± 1.08	0.383 ± 0.05	Unstable
	16.20	48.58	35.22	11.96 ± 0.29	127.5 ± 1.92 ^c^	0.432 ± 0.06	Unstable
	16.20	56.07	27.73	97.43 ± 1.74	28.04 ± 0.02^c^	0.112 ± 0.01	Stable

**^a^** Droplet size significantly (*p* < 0.05) decreased upon increase in concentration of %Smix in the case of NE composition containing OA as oil phase. **^b^** Droplet size significantly (*p* < 0.05) decreased upon increase in concentration of %Smix in the case of NE composition containing C90 as oil phase. **^c^** Droplet size significantly (*p* < 0.05) decreased upon increase in concentration of %Smix in the case of NE composition containing EO as oil phase.

**Table 3:** Effect of mode of mixing of aqueous phase to Oil–S_mix_ phase and vice-versa on OA-based, C90-based, and EO-based NE system.

NE System Composed of OA as Oil to Smix Ratio 1:3 *			
Mode of Mixing	Mean droplet size (nm)	PDI	%T
Instantaneous mixing of aqueous phase to oil–S_mix_ phase	55.47 ± 0.31	0.360 ± 0.031	96.36 ± 0.12
Drop-by-drop mixing of aqueous phase to oil–S_mix_ phase	55.69 ± 0.28	0.261 ± 0.015	96.25 ± 0.04
Instantaneous mixing of oil–S_mix_ phase to the aqueous phase	51.92 ± 0.68	0.248 ± 0.001	96.74 ± 0.25
Drop-by-drop mixing of oil–S_mix_ phase to the aqueous phase	52.29 ± 0.43	0.213 ± 0.014	96.27 ± 0.26
NE system composed of C90 as oil to Smix ratio 1:3 *			
Mode of Mixing	Mean droplet size (nm)	PDI	%T
Instantaneous mixing of aqueous phase to oil–S_mix_ phase	157.49 ± 1.09	0.321 ± 0.012	93.72 ± 0.607
Drop-by-drop mixing of aqueous phase to oil–S_mix_ phase	120.20 ± 1.08	0.324 ± 0.004	95.30 ± 0.188
Instantaneous mixing of oil–S_mix_ phase to the aqueous phase	156.45 ± 0.65	0.321 ± 0.006	93.00 ± 0.445
Drop-by-drop mixing of oil–S_mix_ phase to the aqueous phase	125.51 ± 1.45	0.338 ± 0.007	93.38 ± 0.404
**NE system composed of EO as oil to S_mix_ ratio 1:4 ^#^**			
Mode of Mixing	Mean droplet size (nm)	PDI	%T
Instantaneous mixing of aqueous phase to oil–S_mix_ phase	26.37 ± 0.198	0.145 ± 0.006	98.05 ± 0.035
Drop-by-drop mixing of aqueous phase to oil–S_mix_ phase	26.54 ± 1.126	0.142 ± 0.004	97.98 ± 0.130
Instantaneous mixing of oil–S_mix_ phase to aqueous phase	26.69 ± 0.850	0.133 ± 0.017	97.80 ± 0.531
Drop-by-drop mixing of oil–S_mix_ phase to aqueous phase	27.23 ± 0.145	0.175 ± 0.006	98.18 ± 0.219

* Optimized composition as 300 µL oil, 900 µL Smix phase, and 800 µL water (Supplementary Figure S2). ^#^ Optimized composition as 300 µL oil, 1200 µL S_mix_ phase, and 500 µL water (Supplementary Figure S2).

**Table 4:** Stability study of OA, C90, and EO-based NE system.

Stability Study of NE Composed of Octanoic Acid as Oil Phase at Smix Ratio 1:1										
S. No	O/S Ratio	0 Days			15 Days			30 Days		
		Droplet Size	PdI	%T	Droplet Size	PdI	%T	Droplet Size	PdI	%T
i.	1:1.5	408.64 ± 3.09	0.372 ± 0.051	90.13 ± 0.477	437.94 ± 1.86	0.607 ± 0.021	89.44 ± 1.08	455.51 ± 4.13	0.655 ± 0.029	88.72 ± 0.457
ii.	1:1.8	327.66 ± 3.30	0.366 ± 0.089	86.13 ± 0.320	369.07 ± 16.79	0.478 ± 0.098	84.47 ± 0.480	411.75 ± 10.19	0.572 ± 0.110	91.14 ± 2.10
iii.	1:2	127.86 ± 6.43	0.335 ± 0.013	88.09 ± 0.574	150.64 ± 9.10	0.339 ± 0.019	84.34 ± 0.196	184.39 ± 12.39	0.384 ± 0.017	82.74 ± 0.542
iv.	1:2.5	44.43 ± 0.98	0.077 ± 0.018	97.28 ± 0.196	89.20 ± 2.14	0.230 ± 0.031	94.10 ± 0.111	98.79 ± 3.49	0.229 ± 0.024	94.06 ± 0.075
v.	1:3	50.69 ± 1.88	0.207 ± 0.008	97.54 ± 0.215	52.41 ± 1.10	0.230 ± 0.004	97.29 ± 0.140	54.20 ± 0.439	0.216 ± 0.009	97.14 ± 0.061
vi.	1:3.5	82.02 ± 1.82	0.430 ± 0.016	97.83 ± 0.036	80.26 ± 0.91	0.387 ± 0.012	96.74 ± 0.444	83.62 ± 1.10	0.393 ± 0.004	96.88 ± 0.304
vii.	1:4	96.33 ± 0.30	0.352 ± 0.059	97.55 ± 0.460	95.45 ± 1.17	0.252 ± 0.144	97.37 ± 0.149	97.05 ± 0.538	0.210 ± 0.088	97.10 ± 0.10
**Stability Study of NE Composed of Capryol 90 as Oil Phase at Smix Ratio 1:1**										
S. No.	O/S Ratio	**0 days**			**15 days**			**30 days**		
		Droplet Size	PdI	%T	Droplet Size	PdI	%T	Droplet Size	PDI	%T
i.	1:1.5	329.95 ± 26.62	0.853 ± 0.031	86.74 ± 0.480	677.49 ± 193.3	0.963 ± 0.045	85.56 ± 1.31	742.1 ± 216.7	0.996 ± 0.005	88.21 ± 0.714
ii.	1:1.8	39.88 ± 0.830	0.103 ± 0.001	99.30 ± 0.212	58.27 ± 5.88	0.226 ± 0.026	98.69 ± 0.411	59.24 ± 5.35	0.256 ± 0.015	98.72 ± 0.143
iii.	1:2	51.76 ± 0.364	0.290 ± 0.012	98.60 ± 0.172	70.30 ± 5.01	0.350 ± 0.038	97.65 ± 0.421	73.59 ± 6.83	0.319 ± 0.009	97.85 ± 0.398
iv.	1:2.5	140.24 ± 0.668	0.545 ± 0.003	88.77 ± 0.145	150.05 ± 4.82	0.545 ± 0.040	86.58 ± 0.410	150.89 ± 4.90	0.563 ± 0.015	86.07 ± 0.413
v.	1:3	132.07 ± 1.40	0.367 ± 0.052	92.79 ± 0.061	133.21 ± 2.26	0.337 ± 0.044	92.37 ± 0.223	133.95 ± 1.85	0.230 ± 0.095	92.33 ± 0.144
**Stability Study of NE Composed of Ethyl Oleate as Oil Phase at Smix Ratio 1:1**										
S. No.	O/S Ratio	**0 days**			**15 days**			**30 days**		
		Droplet Size	PdI	%T	Droplet Size	PdI	%T	Droplet Size	PdI	%T
i.	1:2.5	164.07 ± 0.701	0.616 ± 0.009	84.59 ± 0.850	204.59 ± 2.68	0.626 ± 0.059	82.13 ± 1.93	251.32 ± 26.41	0.590 ± 0.019	87.85 ± 3.74
ii.	1:2.8	140.86 ± 0.905	0.128 ± 0.001	83.36 ± 1.49	168.29 ± 3.13	0.217 ± 0.026	81.49 ± 0.405	270.05 ± 17.20	0.295 ± 0.072	80.20 ± 0.234
iii.	1:3	114.92 ± 0.402	0.251 ± 0.009	93.39 ± 0.721	152.12 ± 4.53	0.313 ± 0.027	91.82 ± 0.340	250.14 ± 8.95	0.315 ± 0.052	92.85 ± 0.254
iv.	1:3.5	45.02 ± 3.45	0.549 ± 0.108	89.91 ± 0.461	71.63 ± 4.07	0.618 ± 0.110	90.50 ± 0.125	86.17 ± 9.09	0.549 ± 0.101	90.40 ± 1.38
v.	1:4	27.76 ± 0.276	0.173 ± 0.026	98.88 ± 0.176	27.22 ± 0.199	0.191 ± 0.011	98.81 ± 0.533	27.01 ± 0.115	0.192 ± 0.002	98.46 ± 0.015

## Data Availability

Data are contained within the article.

## Supplementary Materials

The following supporting information can be downloaded at: https://www.mdpi.com/article/10.3390/bioengineering9080384/s1, Figure S1: Schematic illustration presenting formation of nanoemulsion utilizing spontaneous emulsification method; Figure S2: Correlation between %T and mean droplet size of NE system consisting of octanoic acid, capryol 90, and ethyl oleate as oil phase; Figure S3: Optimized composition of nanoemulsion prepared through drop-by-drop mixing of aqueous phase to oil–Smix phase (a) NE system composed of OA as oil having 300 µL oil, 900 µL Smix phase, and 800 µL water, (b) NE system composed of C90 as oil having 300 µL oil, 900 µL Smix phase, and 800 µL water, (c) NE system composed of EO as oil having 300 µL oil, 900 µL Smix phase, and 800 µL water.
