# Supplementary material for: Investigation of Factors Influencing Formation of Nanoemulsion by Spontaneous Emulsification: Impact on Droplet Size, Polydispersity Index, and Stability
Source: Bioengineering (Basel). 2022 Aug 12;9(8):384. doi: 10.3390/bioengineering9080384 (PMC9404776; doi:10.3390/bioengineering9080384)
Supplement: Supplementary file 1 [file bioengineering-09-00384-s001.zip › bioengineering-1825674-supplementary.pdf]

Octanoic acid (Caprylic acid) is a saturated fatty acid with the molecular formula: $C_8H_{16}O_2$, and a molecular weight of 144.21. It consists of an 8-carbon backbone and imparts low lipophilicity.

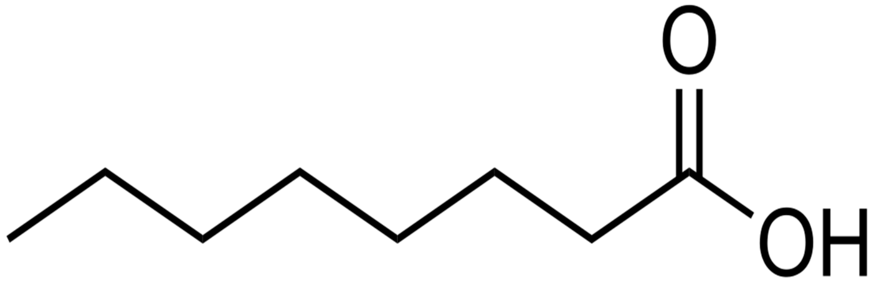

(**Octanoic acid**)

Capryol 90 (1,2-Propylene glycol 1-caprylate) with the molecular formula: $C_{11}H_{22}O_3$, and molecular weight of 202.29. Its molecule consists of an 11-carbon atoms and imparts medium lipophilicity with an HLB value of 5.

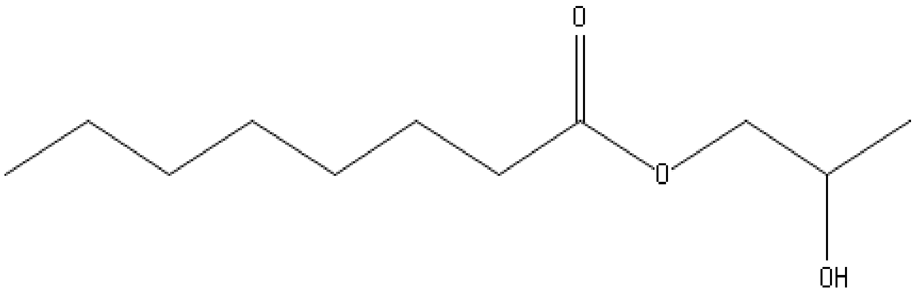

(**Capryol 90**)

Ethyl oleate is a fatty acid ester with the molecular formula: $C_{20}H_{38}O_2$, and a molecular weight of 310.51. It consists of an 18-carbon fatty acid of carbon chain length and imparts high lipophilicity.

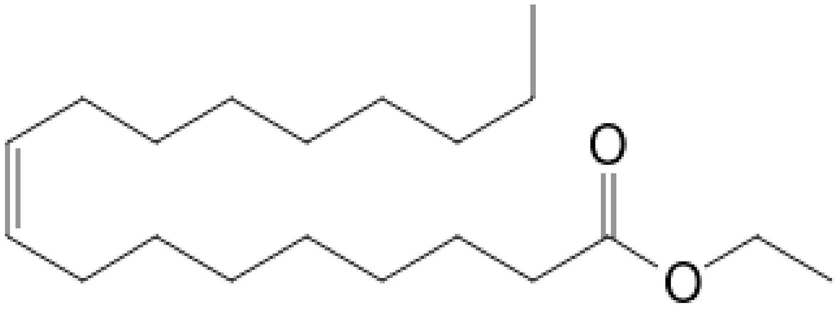

(**Ethyl oleate**)

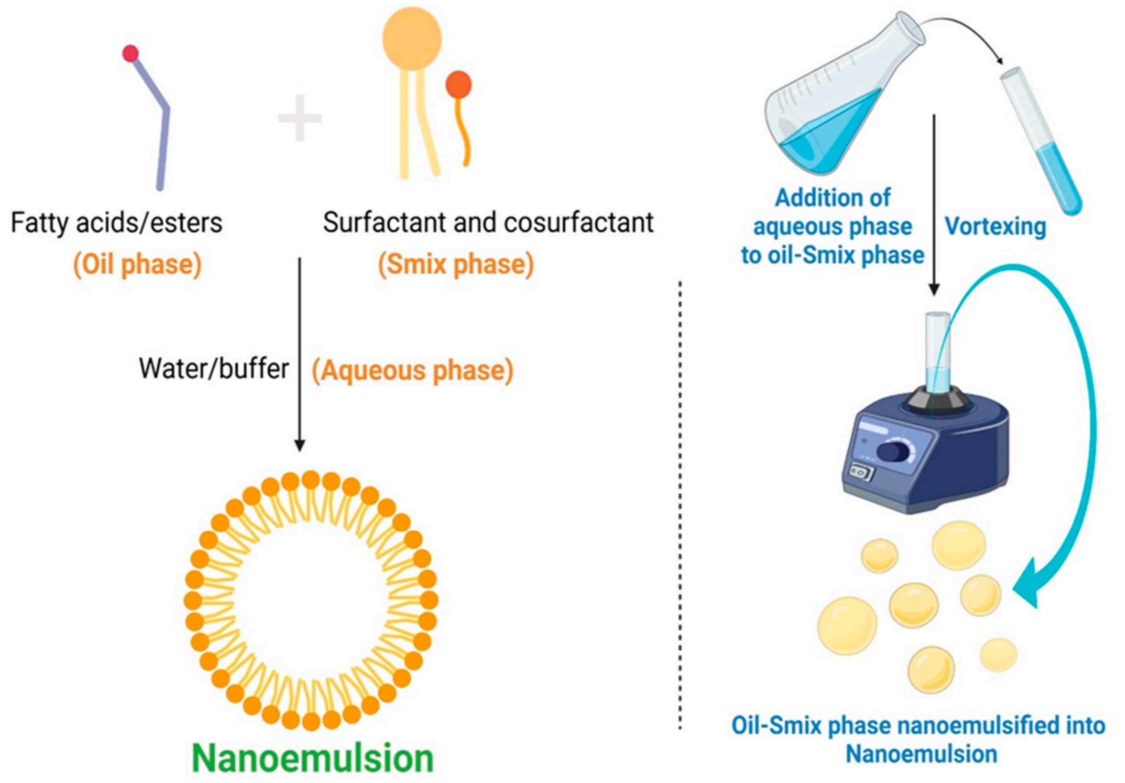

Figure S1. Schematic illustration presenting formation of nanoemulsion utilizing spontaneous emulsification method.

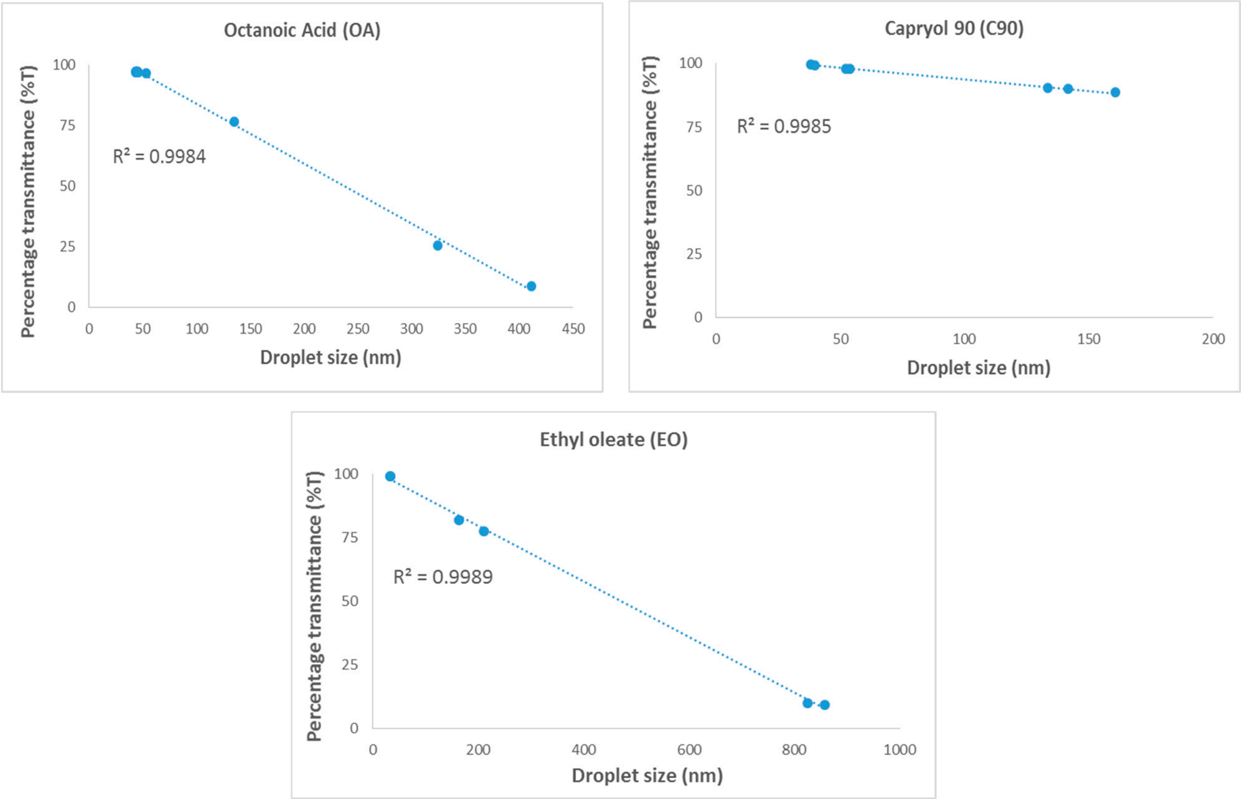

Figure S2. Correlation between %T and mean droplet size of NE system consisting of octanoic acid, capryol 90, and ethyl oleate as oil phase.

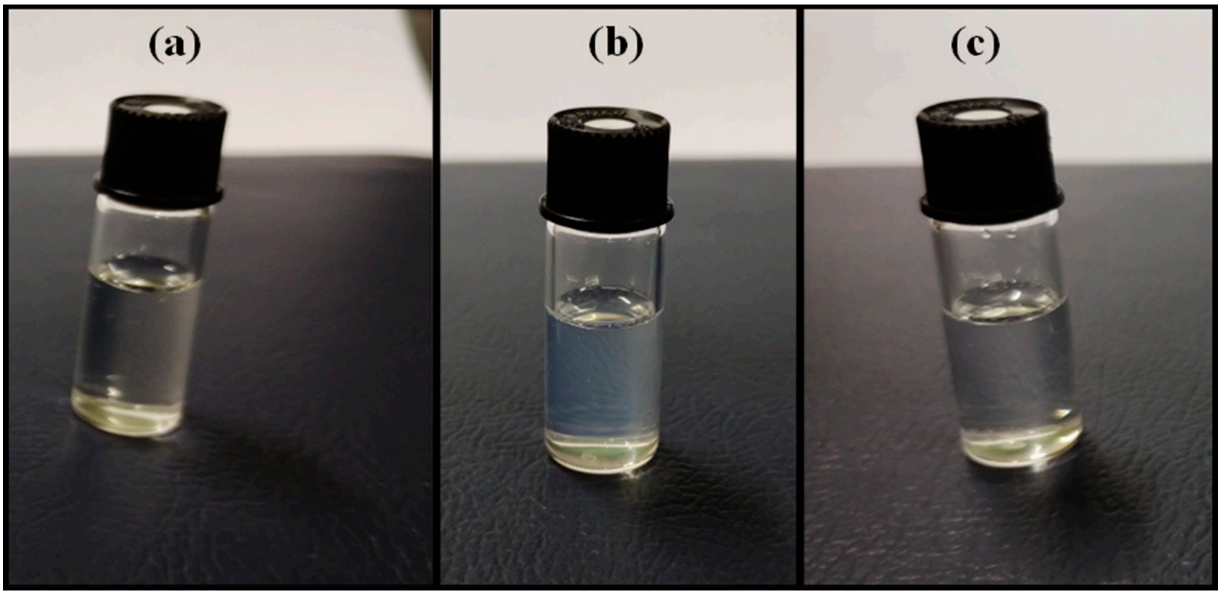

Figure S3. Optimized composition of nanoemulsion prepared through drop-by-drop mixing of aqueous phase to oil-Smix phase (a) NE system composed of OA as oil having 300 µl oil, 900 µl Smix phase, and 800 µl water, (b) NE system composed of C90 as oil having 300 µl oil, 900 µl Smix phase, and 800 µl water, (c) NE system composed of EO as oil having 300 µl oil, 900 µl Smix phase, and 800 µl water.
